# Characterization of a Family with Rare Deletions in *CNTNAP5* and *DOCK4* Suggests Novel Risk Loci for Autism and Dyslexia

**DOI:** 10.1016/j.biopsych.2010.02.002

**Published:** 2010-08-15

**Authors:** Alistair T. Pagnamenta, Elena Bacchelli, Maretha V. de Jonge, Ghazala Mirza, Thomas S. Scerri, Fiorella Minopoli, Andreas Chiocchetti, Kerstin U. Ludwig, Per Hoffmann, Silvia Paracchini, Ernesto Lowy, Denise H. Harold, Jade A. Chapman, Sabine M. Klauck, Fritz Poustka, Renske H. Houben, Wouter G. Staal, Roel A. Ophoff, Michael C. O'Donovan, Julie Williams, Markus M. Nöthen, Gerd Schulte-Körne, Panos Deloukas, Jiannis Ragoussis, Anthony J. Bailey, Elena Maestrini, Anthony P. Monaco, International Molecular Genetic Study Of Autism Consortium

**Affiliations:** a The Wellcome Trust Centre for Human Genetics, University of Oxford, Oxford, United Kingdom; b Department of Biology, University of Bologna, Bologna, Italy; c Department of Child and Adolescent Psychiatry, Rudolf Magnus Institute of Neuroscience, University Medical Center Utrecht, Utrecht, The Netherlands; d Division of Molecular Genome Analysis, German Cancer Research Center, Heidelberg, Germany; e Department of Genomics, Life & Brain Center, University of Bonn, Bonn, Germany; f Institute of Human Genetics, University of Bonn, Bonn, Germany; g MRC Centre for Neuropsychiatric Genetics and Genomics, Cardiff, United Kingdom; h Department of Child and Adolescent Psychiatry, Psychosomatics and Psychotherapy, Goethe-University, Frankfurt/Main, Germany; i Department of Medical Genetics and Rudolf Magnus Institute of Neuroscience, University Medical Center Utrecht, Utrecht, The Netherlands; j University of California Los Angeles Center for Neurobehavioral Genetics, Los Angeles, California; k Department of Child and Adolescent Psychiatry, Psychosomatics and Psychotherapy, Ludwig-Maximilians-University Munich, Munich, Germany; l Wellcome Trust Sanger Institute, Wellcome Trust Genome Campus, Hinxton, United Kingdom; m University Department of Psychiatry, Warneford Hospital, Oxford, United Kingdom

**Keywords:** Autistic, *CNTNAP5*, CNV, *DOCK4*, dyslexia, neurexin

## Abstract

**Background:**

Autism spectrum disorders (ASDs) are characterized by social, communication, and behavioral deficits and complex genetic etiology. A recent study of 517 ASD families implicated *DOCK4* by single nucleotide polymorphism (SNP) association and a microdeletion in an affected sibling pair.

**Methods:**

The *DOCK4* microdeletion on 7q31.1 was further characterized in this family using QuantiSNP analysis of 1M SNP array data and reverse transcription polymerase chain reaction. Extended family members were tested by polymerase chain reaction amplification of junction fragments. *DOCK4* dosage was measured in additional samples using SNP arrays. Since QuantiSNP analysis identified a novel *CNTNAP5* microdeletion in the same affected sibling pair, this gene was sequenced in 143 additional ASD families. Further polymerase chain reaction-restriction fragment length polymorphism analysis included 380 ASD cases and suitable control subjects.

**Results:**

The maternally inherited microdeletion encompassed chr7:110,663,978-111,257,682 and led to a *DOCK4-IMMP2L* fusion transcript. It was also detected in five extended family members with no ASD. However, six of nine individuals with this microdeletion had poor reading ability, which prompted us to screen 606 other dyslexia cases. This led to the identification of a second *DOCK4* microdeletion co-segregating with dyslexia. Assessment of genomic background in the original ASD family detected a paternal 2q14.3 microdeletion disrupting *CNTNAP5* that was also transmitted to both affected siblings. Analysis of other ASD cohorts revealed four additional rare missense changes in *CNTNAP5*. No exonic deletions of *DOCK4* or *CNTNAP5* were seen in 2091 control subjects.

**Conclusions:**

This study highlights two new risk factors for ASD and dyslexia and demonstrates the importance of performing a high-resolution assessment of genomic background, even after detection of a rare and likely damaging microdeletion using a targeted approach.

Autism spectrum disorders (ASDs) are a subset of complex neurodevelopmental disorders characterized by deficits in three core domains: 1) reduced reciprocal social interaction, 2) impaired ability to communicate, and 3) a narrow range of interests and repetitive behaviors. Autism spectrum disorders are clinically heterogeneous and often show comorbidity with other conditions such as epilepsy and learning disability (1,2).

Although autism has consistently been shown to demonstrate high levels of heritability, it has only recently become clear that many of the genes recently implicated in autism are involved with the initiation and maintenance of synaptic connections. For example, a pathway-based analysis using data from the first published ASD genome-wide single nucleotide polymorphism (SNP) association study implicated the cadherin gene family. Stronger enrichment was seen when these 25 cadherins were combined with three neurexins and five neurexin-related *CNTNAP* genes (3). Mutations in neuroligin proteins, which interact with neurexins across the synaptic cleft, were associated with autism before neurexins were implicated (4). Other studies have shown that genomic copy number variations (CNVs) also play a significant role in autism susceptibility (5,6). Hemizygous disruption of the neurexin 1 gene (*NRXN1*) was first seen in sisters with ASD (7). In addition, rare structural variants involving *CNTNAP2* have also recently been associated with autism susceptibility (8,9), further implicating this gene family. Drosophila orthologues of *CNTNAP2* and *NRXN1* can both reorganize synaptic morphology and regulate another synaptic protein bruchpilot (10). It is thus proposed that a shared synaptic mechanism underlies the similar clinical outcomes for patients with *NRXN1* or *CNTNAP2* mutations.

An emerging trend from recent literature is that many microdeletion syndromes exhibit considerable phenotypic variability. For instance, variable dosage of *CNTNAP2* has also been documented in epilepsy and schizophrenia (11), while deletion of 15q13.3 has been implicated in autism (12), generalized learning disability (13), epilepsy (14), and schizophrenia (15). One possible hypothesis is that additional CNVs elsewhere in the genome may act as genetic modifiers. In support of this hypothesis, recent studies on schizophrenia have detected more than one rare CNV > 100 kb within the same affected individual (16).

The autism susceptibility locus 1 (*AUTS1*), situated on chromosome 7q, has been identified in at least four genome-wide linkage scans (17–20) and two meta-analyses (21,22). A recent *AUTS1* fine-mapping study, using both family-based and case-control association analyses, detected SNPs within *DOCK4* (dedicator of cytokinesis 4) and *IMMP2L* (IMP2 inner mitochondrial membrane protease-like) that may be indexing autism susceptibility factors. A rare genomic deletion disrupting both *DOCK4* and *IMMP2L* was also detected and shown to be transmitted to both members of an affected sibling pair (ASP), further implicating this gene region (23). *DOCK4* is a plausible ASD candidate gene, as recent RNA interference studies using rat hippocampal neurons have shown that this gene may influence dendritic branching and growth (24). Genes at this locus have previously been linked to Tourette syndrome, through association (25) or cytogenetic aberration (26). Disruption of a related gene, *DOCK3*, has also been linked to attention-deficit/hyperactivity disorder (27). To understand its potential effect, we first characterized the *IMMP2L-DOCK4* deletion and resulting fusion transcript at base-pair resolution, using a variety of methods. Due to reports of additional neurological and psychiatric disorders in the extended family, relatives for whom DNA was available were also screened for this deletion. The finding that three relatives with poor reading ability had also inherited the same deletion led us to further assess an additional dyslexia cohort for genomic variants in this gene region.

We also assessed the genomic background in the autism family with the *IMMP2L-DOCK4* deletion and detected a second rare microdeletion disrupting *CNTNAP5* in the ASP. As this gene is related to *CNTNAP2*, it was sequenced in additional ASD families from the International Molecular Genetic Study of Autism Consortium (IMGSAC) cohort to search for other rare variants that might be of etiological relevance to autism.

## Methods and Materials

### Clinical Details

The Dutch multiplex autism family 15-0084 volunteered to participate in the IMGSAC research study, as approved by the medical ethical commission of the University Medical Center Utrecht. Extended family members were also asked to participate and donate saliva samples. Informed consent was obtained from all participants. Case reports for the proband (15-0084-003), his affected brother (15-0084-004), and other family members are given in Supplement 1.

### Clinical Assessment

Social communication ability was assessed in all participating family members using the Social Responsiveness Scale (SRS) (28). Depending on the age of the participant, either the children's version or the adult informant version was used. The *t* scores are reported for the SRS children's version. For adults, *t* scores are unavailable, so the total scores are compared with the mean total scores in the Dutch population (Ilse L. Noens, Ph.D., written communication to MVdJ, November 2009). The participants with a formal diagnosis of autism scored within the severe range of the SRS. The total SRS scores of the unaffected family members are all between the mean and +1 SD. Reading and spelling ability were assessed by means of a short battery of Dutch word reading, nonword reading, text reading, and spelling tests. An overall evaluation of communication ability and reading capacity is presented in Table S1 in Supplement 1.

### DNA Extraction

DNA was extracted from the saliva samples of the extended family using the Oragene DNA Extraction Kit (DNA Genotek, Ontario, Canada), following the manufacturer's protocol. DNA from family 15-0084 had been extracted, as described previously (18).

### 1M SNP Array and CNV Analysis

A total of 750 ng of genomic DNA was run on the single-sample Infinium 1M SNP BeadArray (Illumina, San Diego, California) according to manufacturer's instructions, with default SNP clustering. Log R ratios and B allele frequencies were used to call CNVs using the QuantiSNP algorithm (29), with L = 2M, expectation-maximization iterations = 50, MaxCopy = 4, and correcting for local GC content. High confidence calls with log Bayes Factor > 10 were used, whereby we expected approximately one false CNV call per sample.

### Long-Range Polymerase Chain Reaction, Quantitative Multiplex PCR of Short Fluorescent Fragments, and CNV Validation

Long-range polymerase chain reaction (PCR) was carried out with Bio-X-ACT long DNA polymerase (Bioline, London, United Kingdom) using the manufacturer's suggested protocol. Primers TTTCACCTTTTGGGGTGCTA and TGGAGCCTGGGAATTAAAAA were used to amplify across the *IMMP2L-DOCK4* deletion. Nested *CNTNAP5* primer sequences are available on request.

Quantitative Multiplex PCR of Short Fluorescent Fragments (QMPSF) was performed as previously described (30), using DNA from 197 multiplex IMGSAC families (320 affected individuals) and 461 Caucasian United Kingdom control subjects available from the European Collection of Cell Cultures. The QMPSF primers (sequences available on request) were designed in *DOCK4* exons 1, 8, 15, 25, 31, and 52 to complement our previous QMPSF analysis, which included *IMMP2L* exons and the last exon of *DOCK4* (23). Exon 7 of the *RNF20* gene was co-amplified as a control exon.

To ensure that all reported CNV results were verified by at least two methods, DNA samples from extended family members were also tested using quantitative PCR and/or 44 k array-based comparative genomic hybridization (Agilent, Santa Clara, California).

### Real-Time-PCR

Total RNA was extracted from blood samples stored in RNA*later* solution using RiboPure Blood Kit (Ambion, Austin, Texas), according to the manufacturer's instructions. Complementary DNA (cDNA) was synthesized from 500 ng of total RNA using the Superscript III First Strand Synthesis SuperMix for quantitative real-time PCR (qRT-PCR) and random hexamers (Invitrogen, Eugene, Oregon). Semi-quantitative PCR was performed on 1/20th of the cDNA template prepared as described above, with two primer pairs specific for *DOCK4* (available on request) and the third designed to amplify the fusion transcript (*IMMP2L*-x4fus-F: ATCTCCTTCAAGAGCAATCACT; *DOCK4*-x26fus-R: TCTCCTGTCCCTATTACGACAAA), using standard PCR conditions. The PCR was repeated using an increasing number of cycles (from 25 to 40). The PCR products were run on 2% agarose gels in the presence of GelRed (Biotium, Hayward, California). *DOCK4* expression was also evaluated by qRT-PCR using SYBR-green (Applied Biosystems, Foster City, California) with two *DOCK4* specific primer pairs and the housekeeping gene *GUSB* as a control gene. Amplification of serial cDNA dilutions was performed to calculate PCR efficiencies. For each sample, expression was measured in triplicate, with mean values used for calculations. Quantification was calculated using the comparative *C*_T_ method (31).

### NeuroDys Cohort and Additional Control CNV Data

DNA from 232 probands from the NeuroDys dyslexia cohort (http://www.neurodys.com), 643 unrelated Dutch control subjects (32), and 1448 control subjects from the British 1958 Birth Cohort (http://www.b58cgene.sgul.ac.uk) were run on the Infinium 550 K SNP BeadArray (Illumina), according to manufacturer's instructions. Another 374 cases from the NeuroDys cohort were run on the Infinium 300K SNP BeadArray (Illumina). Copy number variation calling was carried out, as described above. Samples were collected following informed consent. The study was approved by the relevant ethics committees.

### CNTNAP5 Sequencing and Bioinformatic Analysis

Polymerase chain reaction products containing all 24 *CNTNAP5* exons and intron-exon boundaries were purified using Exonuclease I (NEB, Ipswich, Massachusetts) and shrimp alkaline phosphatase (USB, Cleveland, Ohio). Sanger sequencing was carried out using BigDye v3.1 (Applied Biosystems). Primers for exons 14, 18, and 22 are listed below; other primer sequences are available on request. Sample selection strategy is presented in Supplement 1. Sequence was analyzed with reference to *CNTNAP5* messenger RNA, NM_130773. Bioinformatic analysis of novel nonsynonymous variants was carried out using PolyPhen (http://genetics.bwh.harvard.edu/pph/) (33). This tool predicts the likely impact of amino acid substitutions on protein function, using known structure and comparative considerations. Predictions should be interpreted with caution as many known pathogenic mutations come up as benign, suggesting a high proportion of false-negatives with this method.

### PCR-Restriction Fragment Length Polymorphism Screening of Additional Cases and Control Subjects

Exon 14 of *CNTNAP5* was amplified using primers TCCAAGATATTTTCAGCTCAGTTTC and GGGGGAGCTCCAGTTTGTAT and standard PCR conditions. The wild-type 362 base pair (bp) PCR amplicon is cut into 141 bp, 24 bp, and 197 bp fragments by the enzyme *Mnl*I (NEB). One of the CCTC recognition sites is disrupted by the c.2141C > G mutation, resulting in fragments of 141 bp and 221 bp.

Exon 18 was amplified using primers AGACCTTCATGACAACAGAGAACT and AAACAGCGGGAGAGTAGATGA. The enzyme *Hpy*CH4IV (NEB) cuts the wild-type 469 bp amplicon into 119 bp and 350 bp fragments. However, the c.2756C > T mutation disrupts this ACGT recognition site.

Exon 22 was amplified with primers GGAGGTGGGGAAAGGAGATA and AAAAACTGAGTCACATTAGCCAAC. The c.3502G > A mutation creates a *Bcc*I (NEB) restriction site (CCATC) in the middle of the 490 bp amplicon resulting in 242 bp and 248 bp fragments, while the c.3584C > T polymorphism creates an alternative restriction site, resulting in fragments of 179 bp and 311 bp.

All digestion products were visualized on 1.8% agarose gels, stained with SYBR safe (Invitrogen). Caucasian control samples included Human Random Control panels available from the European Collection of Cell Cultures, additional German control DNAs, and also DNA from parents of anorexia patients. In addition, 380 unrelated German ASD patients (34) were screened for the c.2756C > T and c.3502G > A variants.

## Results

**Figure 1:**
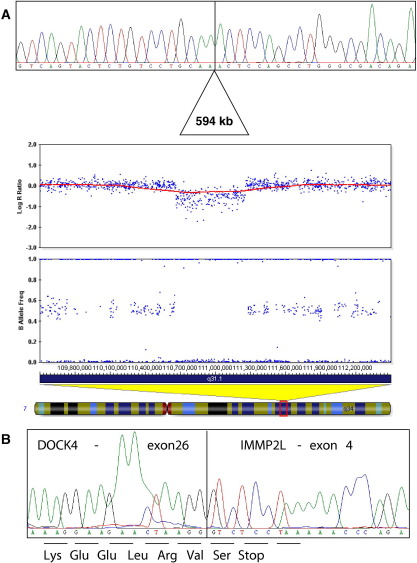
Molecular characterization of the *IMMP2L-DOCK4* deletion. **(A)** Shows sequence electropherogram from the long-range PCR product spanning the deletion, aligned with the BeadStudio plots of the log R ratio and B allele frequency data from the 1M SNP array for 15-0084-003. Red plot indicates 1 Mb moving-window average of the log R ratio across the region chr7:109.5-112.5 Mb. **(B)** Shows sequence from RT-PCR product showing evidence of a transcript that fuses *DOCK4* exon 26 onto *IMMP2L* exon 4. PCR, polymerase chain reaction; RT-PCR, real-time-polymerase chain reaction; SNP, single nucleotide polymorphism.

In a recent CNV scan of the *AUTS1* locus, we detected a ∼ 600 kilobase (kb) deletion involving *IMMP2L* and *DOCK4* in an autistic ASP. Quantitative PCR had been used to show that the distal breakpoint lay between exons 14 and 31 of *DOCK4* (23). To further resolve the deletion at both extremities, DNA samples from the family were run on 1M SNP arrays. Over 1M SNPs were called for all five family members, with low rates of Mendelian error (Table S2 in Supplement 1). Using QuantiSNP, the *IMMP2L-DOCK4* deletion was the most confidently detected CNV, with a log Bayes Factor of 777 in proband 15-0084-003 (Table 1 and Table S3 in Supplement 1). These array data were consistent with our previous studies that showed that the deletion had been transmitted from the reading-impaired mother (15-0084-002) to all three children (two boys with autism and their reading-impaired sister). However, as the QuantiSNP CNV calls were inconsistent in terms of deletion size, visual inspection of the log R ratio and B allele frequencies within BeadStudio software (Illumina) allowed us to determine that the proximal deletion breakpoint lay between rs37713 and rs37715, while the distal breakpoint was situated between rs6966622 and rs10238664. This resolution was sufficient to allow the design of suitable long-range PCR primers. Amplification across the deletion breakpoint followed by Sanger sequencing allowed precise characterization of the 594-kb deletion (Figure 1A). The ACTCYAGCC motif observed at either end of the chr7:110,663,978-111,257,682 deletion (National Center for Biotechnology Information build 36 coordinates) suggested that some sequence similarity-driven process may have originally led to this deletion.

While *IMMP2L* shows variable dosage at a similar rate in autism cases and in control subjects (23), there were no exonic deletions of *DOCK4* listed in the Database of Genomic Variants (DGV) (March 2009 release, http://projects.tcag.ca/variation/) (35). We assessed the frequency of *DOCK4* CNVs in 197 additional multiplex IMGSAC autism families (320 affected individuals) and 461 Caucasian control subjects using six exonic QMPSF probes. No additional *DOCK4* deletions or duplications were seen in ASD families, besides the deletion in family 15-0084 described above and the duplication in family 13-3023 reported previously (23). No CNVs were seen in the control subjects.

Recent studies have shown that deletions that extend from one gene to the next can lead to potentially deleterious fusion transcripts (16). As *IMMP2L* and *DOCK4* are transcribed in the same direction, we assessed whether the presence of the deletion results in a fusion transcript. Indeed, RT-PCR and Sanger sequencing detected a fusion transcript that was not present in control RNA. The *IMMP2L* section of the *DOCK4*-*IMMP2L* fusion transcript is out of frame. Only two novel amino acids (Valine-Serine) would be translated, immediately followed by a premature stop codon (Figure 1B). It is thus likely to be subject to nonsense-mediated-decay, consistent with semiquantitative RT-PCR data suggesting that this fusion transcript was present at low levels in relation to the wild-type transcript. Expression of the normal *DOCK4* transcript was evaluated by qRT-PCR in the mother with the deletion and found to be 30% to 50% lower compared with the father with normal copy number.

**Figure 2:**
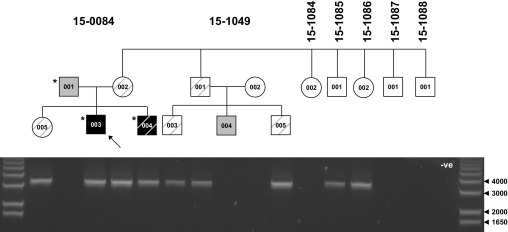
Inheritance pattern of the *IMMP2L-DOCK4* deletion within the extended family. Long-range PCR products of 3087 bp are visible only where this deletion is present. Gel lanes are aligned with the pedigree, with proband indicated by an arrow. The size of relevant bands in the DNA ladder are indicated in base pairs. Dark shading indicates ADI-defined autism, lighter shading indicates Asperger syndrome or autistic features, and diagonal stripes indicate dyslexic diagnosis or reading impaired. Asterisk indicates presence of *CNTNAP5* deletion. ADI, Autism Diagnostic Interview; bp, base pair; PCR, polymerase chain reaction.

Long-range PCR was used to screen the mother's extended family for this 594 kb *IMMP2L-DOCK4* deletion. The presence of the deletion in the proband's brother, sister, mother, uncle, and two cousins, all of whom performed poorly on reading assessment (Figure 2, Table S1 in Supplement 1), suggested that this deletion may also act as a risk factor for dyslexia. These findings led us to assess the NeuroDys cohort comprising 606 unrelated individuals with dyslexia that were tested for CNVs using Infinium SNP arrays. There was a deletion of *DOCK4* exons 38 to 52 (detected on the 300K array, log Bayes Factor = 44, validated by QMPSF and quantitative PCR) in a single dyslexia case, but no exonic deletions of *DOCK4* were detected in 2091 control samples tested on the 550 K SNP array (*p* = .225, Fisher's exact test). This 132 kb deletion was transmitted from a father reported to have had dyslexia in childhood. Both the father and son presented with slow reading speed. The unaffected sister had not inherited the deletion.

**Figure 3:**
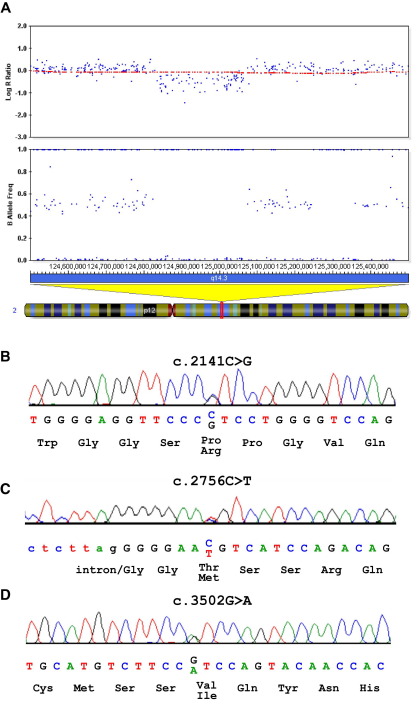
Molecular analysis of *CNTNAP5*. **(A)** Shows the log R ratio and B allele frequency 1M SNP data for 15-0084-003. Red plot indicates 1 Mb moving-window average of the log R ratio across the region chr2:124.5-125.5 Mb. **(B–D)** Shows sequence electropherograms of the three novel nonsynonymous variants detected in *CNTNAP5*. SNP, single nucleotide polymorphism.

To assess the genomic context of the *IMMP2L-DOCK4* deletion in family 15-0084, all 43 CNVs detected in the proband with log Bayes Factor > 10 were analyzed. While most other CNVs were well represented in the DGV, one on chromosome 2 was notable (Figure 3A). This was the second highest ranking CNV, detected with log Bayes Factor of 313 (Table 1 and Table S3 in Supplement 1). This 227 kb deletion removes exons 4 to 11 of *CNTNAP5*. There were no exonic deletions of this gene listed in the DGV or in 2091 control samples tested on the 550K SNP array. Nested long-range PCR was used to validate this deletion and resolve the breakpoints to 6 kb LINE-1 elements. Segregation analysis indicated that this deletion had been transmitted from the father to both autistic sons but not to their dyslexic sister (Figure 2, Table 1).

Interestingly, the father with the *CNTNAP5* deletion was reported to exhibit various autistic traits, although he declined formal testing. In addition, various neuropsychiatric traits were described in members of his extended family, including eight others with reported impairments in social communication. We hypothesized that these relatives may have inherited the same deletion of *CNTNAP5*. However, DNA was only available for the proband's paternal aunt (who was reported to have schizophrenia) and she did not carry the *CNTNAP5* deletion.

*CNTNAP5* transcripts missing exons 4 to 11 are predicted to result in a frameshift, with 54 novel amino acids followed by premature termination codon but also probably inducing nonsense-mediated-decay. However, *CNTNAP5* was undetectable in blood RNA by RT-PCR, so it was not possible to confirm the functional effect of this exonic deletion.

As there are no disruptions of *CNTNAP5* reported in eight recent whole genome CNV scans listed in the Autism Chromosome Rearrangement Database (http://projects.tcag.ca/autism, December 2009 update), we decided to further assess the potential role of rare *CNTNAP5* variants in ASD, by sequencing all 24 exons and intron-exon boundaries for probands from 143 ASD families. Three nonsynonymous mutations were detected in Caucasian ASD probands that were not present in dbSNP build 130 (36). The first was a c.2141C > G transversion in exon 14 that predicts a proline to arginine change at position 714 (P714R) of the amino acid sequence (Figure 3B) in the fibrinogen C-terminal domain. This mutation was maternally transmitted to both affected sons but not to their unaffected sister (Figure S1A in Supplement 1). Although position 714 (P714R) is not conserved through evolution (bioinformatics analysis predicted this change to be benign), the loss of a proline residue may alter protein structure. Using PCR-restriction fragment length polymorphism (RFLP), this mutation was detected in only 1/932 Caucasian control chromosomes. The second mutation was a c.2756C > T transition in exon 18 that predicts a threonine to methionine (T919M) change (Figure 3C) in the laminin G-like 3 domain. This mutation, found in a German family, was maternally transmitted to both affected children but not to their two unaffected siblings (Figure S1B in Supplement 1). Based on the alignment of homologous protein sequences around position 919, bioinformatic analysis predicted that this amino acid substitution is likely to have a damaging effect. Using PCR-RFLP, this variant was not detected in 1222 Caucasian control chromosomes, of which 660 were German. The third mutation was a c.3502G > A transition in exon 22 that predicts a valine to isoleucine (V1168I) change (Figure 3D) within the laminin G-like 4 domain. This mutation, found in another German family, was maternally transmitted to both affected sons but not to their unaffected brother (Figure S1C in Supplement 1). Although this is a relatively minor amino acid change (both are aliphatic residues and hence bioinformatic analysis predicted this change to be benign), V1168I is highly conserved across species. The PCR-RFLP assay also did not detect this change in 1232 control chromosomes, of which 692 were German.

The two changes that were not detected in control subjects (T919M and V1168I) were further assessed by PCR-RFLP in a cohort of 380 unrelated German ASD patients. While no additional cases of T919M were detected, the V1168I mutation was seen in a second German ASD case. Therefore, in total, this change was found in 2 of 523 ASD probands and 0 of 616 control subjects (*p* = .211). However, in this second family, the mutation had not been transmitted to an affected brother (Figure S1D in Supplement 1).

The exon 22 RFLP assay for V1168I was also able to detect a c.3584C > T transition polymorphism that predicts a threonine to methionine (T1195M) change. In total, this polymorphism (rs34165507) was detected at a 3.1% minor allele frequency in ASD cases (32 of 1046 chromosomes) compared with 2.9% in control subjects (36 of 1232 chromosomes).

Finally, DNA from the ASD proband 15-0084-003 was also sequenced to determine whether the paternally inherited *CNTNAP5* deletion was potentially unmasking maternally inherited variants in the same gene. However, only a novel T > C change in a nonconserved region of the 5′-UTR and another common intronic polymorphism (rs924802) were detected, neither of which are likely to affect gene expression or function.

## Discussion

In our earlier study, coincident SNP association and CNV data in *AUTS1* implicated the *IMMP2L-DOCK4* gene region in ASD susceptibility (23). In contrast, another study using 7q-linked ASD families did not see association in this gene region (37). This discrepancy may be because the two studies used different markers. In addition, the negative study may have been underpowered, as only 30 families were used and the markers density was insufficient for CNV analysis to be performed.

In the study described here, characterization of the 594 kb deletion described earlier (23) using a high-density genome-wide microarray has led to the discovery of a second rare deletion within the same affected individuals disrupting the *CNTNAP5* gene. We hypothesize that both CNVs contribute to the array of phenotypes seen in family 15-0084. This study thus demonstrates the importance of performing a high-resolution assessment of genomic background, even after the detection of a rare and likely damaging CNV using a targeted approach.

The rare 594 kb deletion results in an *IMMP2L-DOCK4* fusion transcript. Future studies should determine whether this is a common property of deletions. The deletion was also detected in the affected boys' sister, mother, and five members of their extended family. Although only 15-0084-004 had been formally diagnosed as being dyslexic, the finding that the reading abilities of 6 of 9 individuals with the deletion were weak or very weak, and two other individuals were in the low-average range (Table S1 in Supplement 1) led us to further assess the potential role of *DOCK4* deletions in dyslexia. Another *DOCK4* deletion was detected in one case that showed co-segregation with dyslexia within the nuclear family. Combining QMPSF and 550 K SNP array data was consistent with the DGV, indicating *IMMP2L* dosage to be variable in control subjects, while exonic deletions of *DOCK4* were undetectable in > 2500 control subjects. In contrast to recent CNV studies on attention-deficit/hyperactivity disorder (38), our data therefore suggest that haploinsufficiency of *IMMP2L* might be benign, with deletions of *DOCK4* more likely to be of etiological relevance. Although larger studies would be required to reach formal significance and confirm the relevance of *DOCK4* to reading ability, it is worth noting that 1) case-control analysis does not consider the segregation seen, and 2) a previous study of 11 large Finnish dyslexia pedigrees detected linkage at 7q32 (maximum nonparametric genome-wide linkage score of 2.77) 17.4 Mb distal to *DOCK4* (39).

*DOCK4* functions as a guanine nucleotide exchange factor (GEF), which activates both Rap1 and Rac1 small guanosine triphosphate (GTP)ases by exchanging bound guanosine diphosphate for free GTP (40) and thus acts as a positive regulator of dendritic growth in neuronal cell lines (24). Interestingly, a previous study found five families with rare nonsynonymous variants in another GEF (cAMP-GEFII), which co-segregated with ASD (41). Further studies to elucidate the role of *DOCK4* in human neuronal cell lines and more generally the role of GEF regulators of small GTPases in autism and dyslexia are now warranted.

There are five members of the human contactin-associated protein family (CNTNAP1–5). These transmembrane proteins are predominantly expressed in the central nervous system and are thought to be involved in cell recognition and adhesion. Of the five, *CNTNAP5* is perhaps the least well-studied. However, null mutations of one of the three mouse orthologs (Caspr5-2) result in perinatal lethality, while heterozygous animals show no obvious phenotype (42). A nonsynonymous SNP in *CNTNAP5* was recently identified in a genome-wide pharmacogenomic study as potentially influencing the effect of the drug risperidone on negative symptoms in schizophrenia (43). Only a single human case with deletion of *CNTNAP5* has been described previously (44). This patient harbored a complex de novo chromosomal rearrangement involving chromosomes 1, 2, and 15 and exhibited learning disability and language impairment but did not meet the criteria for ASD. Fine-mapping studies using fluorescence in situ hybridization and array-based comparative genomic hybridization indicated that the complex rearrangement involved eight separate breakpoints. Although the authors suggest that the cryptic deletion of *CNTNAP5* may underlie the phenotype seen in this patient, the complexity of the genomic rearrangement complicates interpretation.

Given the role of related neurexin genes in synapse function, the absence of exonic deletions of *CNTNAP5* from 2091 control subjects, and the deletion's co-segregation with ASD in family 15-0084, we hypothesized that other rare variants in this gene may influence ASD susceptibility. To examine this possibility, sequencing was carried out in 143 multiplex families. The co-segregation with ASD seen in three of the four families and the scarcity of the three novel missense mutations in control subjects is consistent with rare variants of this gene playing a role in ASD susceptibility. The *CNTNAP5* analysis undertaken here has only included multiplex ASD families. This type of family is less likely to harbor de novo ASD risk factors than are sporadic ASD cases (6). Further studies into the potential role of *CNTNAP5* in ASD should use sporadic cases to complement the work described here. Additional studies should also help determine the effect of these mutations on synapse function.

In conclusion, our data suggest that exonic deletions of *DOCK4* may act as a risk factor for reading impairment while rare variants in *CNTNAP5* may confer ASD susceptibility. Genomic disruption of both genes may have an additive effect and in this family appears to result in a more severe ASD phenotype. These data add to the emerging theme that the same genomic variants, in combination with distinct genetic backgrounds, may contribute to different phenotypes.

## Figures and Tables

**Table 1:** QuantiSNP Data for the *IMMP2L-DOCK4* and *CNTNAP5* Gene Loci

Sample ID	Chr	Start (bp)	End (bp)	Length (bp)	Start	End	Number SNPs	Copy Number	Log Bayes Factor	Gene(*s*)
15-0084-002	7	110,654,771	111,256,808	602,037	rs214475	rs6966622	179	1	543.06	*DOCK4, IMMP2L*
15-0084-003	7	110,666,487	111,256,808	590,321	rs37715	rs6966622	175	1	777.48	*DOCK4, IMMP2L*
15-0084-004	7	110,254,457	111,266,360	1,011,903	rs10279573	rs7783121	310	1	169.92	*DOCK4, IMMP2L*
15-0084-005	7	110,247,163	111,266,360	1,019,197	rs1569122	rs7783121	311	1	208.85	*DOCK4, IMMP2L*
15-0084-001	2	124,836,663	125,063,827	227,164	rs11688892	rs4848944	84	1	117.45	*CNTNAP5*
15-0084-003	2	124,836,663	125,063,827	227,164	rs11688892	rs4848944	84	1	313.38	*CNTNAP5*
15-0084-004	2	124,829,445	125,111,265	281,820	rs7578650	rs11900792	94	1	60.77	*CNTNAP5*

Physical positions shown are according to National Center for Biotechnology Information Build 36. bp, base pair; Chr, chromosome; SNP, single nucleotide polymorphism.
