# Supplementary material for: Characterization of a Family with Rare Deletions in CNTNAP5 and DOCK4 Suggests Novel Risk Loci for Autism and Dyslexia
Source: Biol Psychiatry. 2010 Aug 15;68(4):320–8. doi: 10.1016/j.biopsych.2010.02.002 (PMC2941017; doi:10.1016/j.biopsych.2010.02.002)
Supplement: Supplement 1 [file mmc1.pdf]

Supplemental Information

Case reports

Participant 15-0084-003 (proband) was born after a normal pregnancy, weighing 4200 g. He was cyanotic at birth and needed extra oxygen. Language development was delayed, with first words and short phrases at 39 months and severe articulation difficulties at 5 years. He showed no pretend play, but significant stereotyped play, including a special skill for building complex constructions. At the time of assessment, language skills were age-appropriate but intonation was flat and social communication markedly limited. He was socially withdrawn and resistant to change. He attended special education and had reading difficulties. He received a clinical diagnosis of autism (DSM-IV) and met criteria for autism on the Autism Diagnostic Interview-Revised (ADI-R) (1) and the Autism Diagnostic Observation Schedule (ADOS) (2).

Participant 15-0084-004 was born after a normal pregnancy, weighing 3950 g. Language development was delayed (first words 30 months; phrases 42 months). He used stereotyped language and reciprocity in communication or social interaction was markedly limited. He showed no pretend or social play. At the time of assessment, his language skills were age-appropriate and reciprocity in communication was improved. Eye contact and facial expression were markedly limited. He showed ritualistic behavior, stereotyped hand and finger movements and resistance to change. He was clinically diagnosed with autism (DSM-IV) and met criteria for autism on the ADI-R and ASD on the ADOS. Despite a normal intelligence, he was also diagnosed with dyslexia and so receives special education. A normal karyotype was observed and molecular testing for Fragile X was negative.

Participant 15-0084-005 was born without complications after a normal pregnancy, weighing 3600 g. Early development was normal and there were no concerns about her social, communication or cognitive development or physical health. Despite reading difficulties (no formal assessment or diagnosis of dyslexia), she finished high-school education.

The father (15-0084-001) is one of 6 children. Family history revealed fibromyalgia, breast cancer and schizophrenia among his siblings. Several family members (including the father) have social or communication difficulties; however, none have undergone formal psychiatric assessment. Among the children of the father's siblings, there are several with social communication or learning difficulties and/or with symptoms or a diagnosis of autism.

The mother (15-0084-002) is one of 8 children. Three other children died at birth as a result of prematurity (twins) and birth complications. Family history revealed rheumatoid arthritis and kidney dysfunction. The mother reported no difficulties in social interaction, communication ability or behavioral problems, in herself or her siblings. However, there is a family history of reading and spelling difficulties. The mother has problems with reading and spelling (although there is no formal diagnosis of dyslexia). Her brother (15-1049-001) and two of his sons (15-1049-003 and 15-1049-005) also experience significant problems with reading and spelling. Another son of 15-1049-001 was diagnosed with Asperger disorder, aged 20.

The pedigree of this family is presented in Figure 2.

Sample- selection strategy for CNTNAP5 sequencing

Only 1 affected individual per family was selected for sequencing. From a total of 262 multiplex IMGSAC ASD families, we prioritized 143 based on non-parametric linkage (NPL) analysis per family using Merlin (www.sph.umich.edu/csg/abecasis/Merlin), using data from a previous study (3). Families for whom ASPs did not share either copy of *CNTNAP5* identical-by-decent (IBD) were removed from this sequencing effort, as it was reasoned that such families were unlikely to harbor ASD-susceptibility variants in this gene. In total, we estimate that for ~40% of families sequenced, ASPs shared both copies of *CNTNAP5* IBD, compared to the 25% that would be expected in unselected populations.

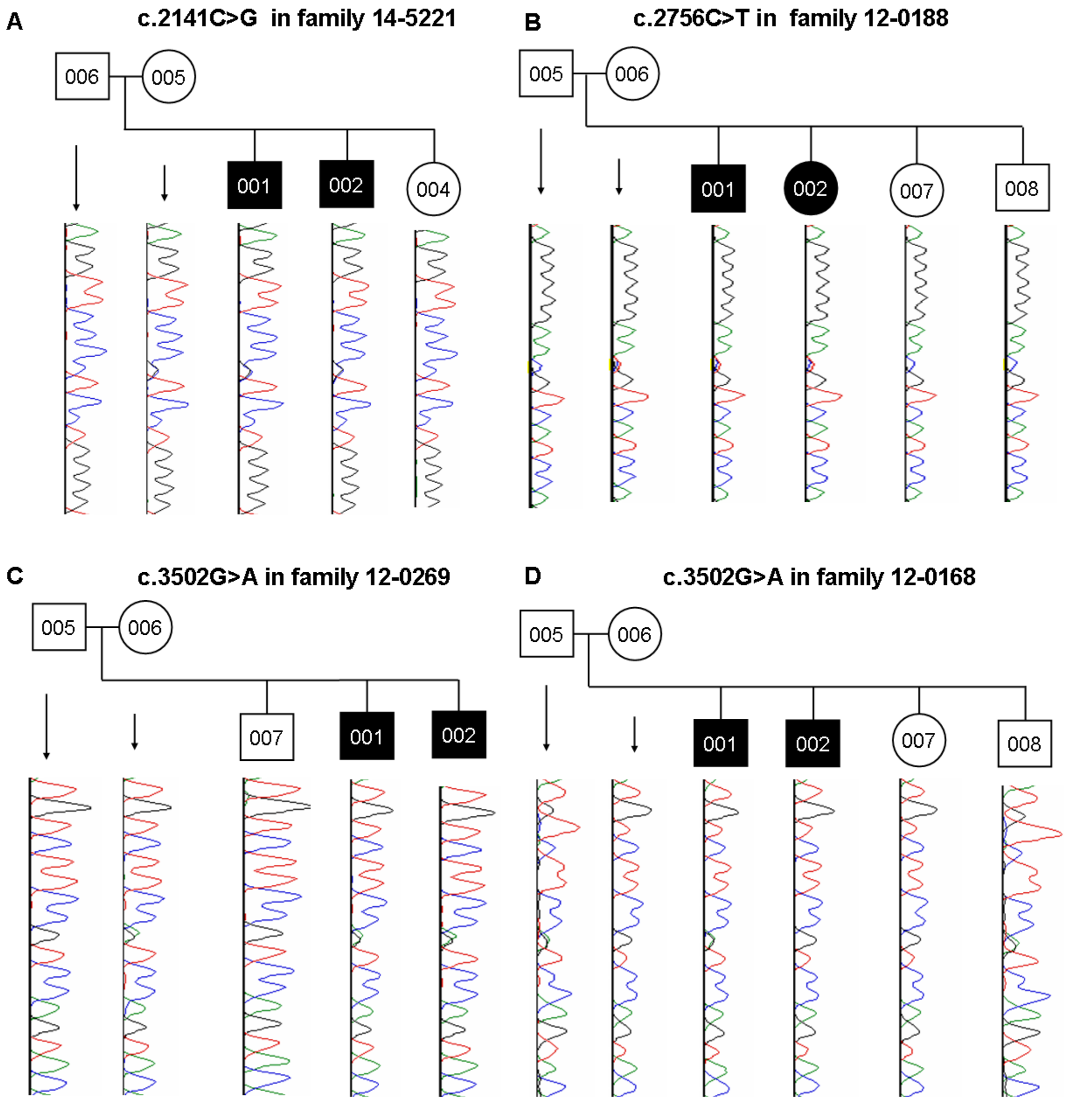

Figure S1. Sequencing electropherograms showing the segregation pattern of the three novel non-synonymous changes identified in *CNTNAP5*. Shaded symbols indicate ASD. All results were also confirmed with PCR-RFLP.

Table S1. Phenotype summary of individuals carrying *IMMP2L-DOCK4* deletion or *CNTNAP5* deletion

Family member	Gender	Deletion status	Clinical report	Social responsiveness	Reading results summary	Verbal PPVT-III-NL score	Performance RAVEN SPM score	Highest level education	Occupation
15-0084-001	Male	*CNTNAP5*	Social communication problems	SRS total score 42	Average	-	-	Higher Vocational Education	Professional
15-0084-002	Female	*IMMP2L-DOCK4*	Reading/spelling difficulties	SRS total score 33	Very weak	-	-	Intermediate Vocational Training	None
15-0084-003	Male	*IMMP2L-DOCK4* *CNTNAP5*	Autism (DSM-IV) + reading/spelling difficulties	SRS T-score 91 (severe range)	(Low) average	121	102	General Certificate of Secondary Education C level	None (student)
15-0084-004	Male	*IMMP2L-DOCK4* *CNTNAP5*	Autism (DSM-IV) + dyslexia	SRS T-score 84 (severe range)	Very weak	115	112	Elementary school	None (student)
15-0084-005	Female	*IMMP2L-DOCK4*	Reading/spelling difficulties	SRS total score 41	Very weak	122	90	pre-university high-school education	None (student)
15-1049-001	Male	*IMMP2L-DOCK4*	Reading/spelling difficulties	SRS total score 40	Very weak	-	106	Intermediate Vocational Training	Associate professional
15-1049-003	Male	*IMMP2L-DOCK4*	Reading/spelling difficulties	SRS total score 12	Weak	-	103	Intermediate Vocational Training	None (student)
15-1049-005	Male	*IMMP2L-DOCK4*	Reading/spelling difficulties	SRS total score 28	Very weak	-	110	General Certificate of Secondary Education C level	Skilled manual
15-1085-001	Male	*IMMP2L-DOCK4*	-	SRS total score 19	(Low) average	105	108	Higher Vocational Education	Professional
15-1086-002	Female	*IMMP2L-DOCK4*	-	SRS total score 29	Average	97	92	General Certificate of Secondary Education C level	Skilled manual

SRS, Social Responsiveness Scale; PPVT-III-NL, Peabody Picture Vocabulary Test, 3rd edition; SPM, Standard Progressive Matrices

Table S2. SNP quality control data from 1M BeadArray

Sample ID	Calls	No Calls	Call Rate	10% GC Score	Correct inheritance	Mendelian errors	P-P-C Heritability
15-0084-001	1,033,134	15,874	0.985	0.614	N/A	N/A	N/A
15-0084-002	1,041,857	7,151	0.993	0.638	N/A	N/A	N/A
15-0084-003	1,046,915	2,093	0.998	0.649	1,017,702	8,046	0.992
15-0084-004	1,008,935	40,073	0.962	0.556	984,754	8,949	0.991
15-0084-005	1,016,594	32,414	0.969	0.577	1,000,735	880	0.999

Table S3. QuantiSNP CNV calls for proband 15-0084-003. All CNVs with log Bayes Factor > 10 are listed in order of how confidently they were detected. *crosses centromere

Chr	Start (bp)	End (bp)	Length (bp)	Start	End	No. SNPs	Copy Number	Log Bayes Factor	Gene(s)
7	110,666,487	111,256,808	590,321	rs37715	rs6966622	175	1	777.48	*IMMP2L, DOCK4*
2	124,836,663	125,063,827	227,164	rs11688892	rs4848944	84	1	313.38	*CNTNAP5*
11	92,396,199	92,543,215	147,016	rs9971402	rs10830997	48	1	218.91	*SLC36A4*
7	141,412,174	141,439,888	27,714	rs4276595	rs4726489	15	0	104.08	*MGAM*
15	28,294,753	28,580,907	286,154	rs4043159	rs2648193	26	1	87.67	*CHRFAM7A*
6	67,074,215	67,105,019	30,804	rs7773946	rs1634207	21	1	80.44	-
6	32,587,470	32,664,508	77,038	rs35847514	rs35283503	51	1	69.28	*HLA-DRB5, HLA-DRB6, HLA-DRB1*
9	138,507,667	138,737,806	230,139	rs9314867	rs7041513	96	3	61.54	*NOTCH1, EGFL7, hsa-mir-126, AGPAT2, FAM69B*
6	79,029,649	79,090,197	60,548	rs818248	rs7773124	33	1	55.04	-
3	192,548,086	192,552,678	4,592	rs6444540	rs297396	8	0	54.07	*CCDC50*
1	121,064,204	142,454,940	21,390,736*	rs35792998	rs35826342	35	1	44.17	-
10	38,778,547	38,930,249	151,702	rs28448770	rs12161668	26	1	41.15	*LOC399744*
8	7,023,889	8,124,711	1,100,822	rs10113807	rs3958844	38	1	38.47	*LOC349196, DEFB109P1B, DEFB103A, DEFB103B, SPAG11B, DEFB104A, DEFB104B, DEFB106A, DEFB106B, DEFB105A, DEFB105B, DEFB107B, DEFB107A, FAM90A7, DEFB4, FAM66E, hsa-mir-548i-3, FLJ10661*
X	154,433,477	154,435,960	2,483	rs6642287	rs5940491	4	0	32.25	*TMLHE*
7	61,792,309	62,035,077	242,768	rs8188311	rs1304186	27	3	30.95	-
8	39,351,896	39,460,558	108,662	rs10108977	rs7830411	17	1	29.91	*ADAM5P, ADAM3A*
11	50,440,511	51,152,280	711,769	rs11561057	rs11522765	50	3	27.64	-
4	39,385,203	39,402,627	17,424	rs7693996	rs13147205	10	1	26.71	*UBE2K*
3	177,371,924	177,390,036	18,112	rs7626997	rs2067613	8	1	24.89	-
4	92,499,956	92,503,958	4,002	rs12507382	rs10516891	4	0	23.98	*FAM190A*
10	58,185,524	58,190,329	4,805	rs12250344	rs12767547	9	1	23.36	-
11	55,124,465	55,200,052	75,587	rs596371	rs17499045	33	1	22.86	*OR4C11, OR4P4, OR4S2, OR4C6*
1	3,364,220	3,447,155	82,935	rs12745860	rs947347	30	3	18.45	*ARHGEF16, MEGF6*

1	202,176,843	202,184,893	8,050	rs1935588	rs12065650	6	1	17.66	-
7	1,518,242	1,560,489	42,247	rs10282181	rs10155977	22	4	17.31	*MAFK, TMEM184A*
9	66,464,433	67,055,399	590,966	rs2321402	rs12552652	20	1	16.89	*AQP7P1, AQP7P2*
22	37,693,776	37,711,772	17,996	rs6001344	rs2076109	8	1	15.67	*APOBEC3B*
21	9,730,102	9,849,624	119,522	rs4044122	rs28970308	10	1	14.80	-
2	146,583,025	146,592,386	9,361	rs12465919	rs222833	3	0	14.16	-
6	32,574,137	32,577,503	3,366	rs28877027	rs28895902	2	0	13.36	-
2	91,546,258	91,672,122	125,864	rs12997258	rs4509760	6	1	13.24	-
12	34,320,124	34,435,946	115,822	rs11053185	rs11831993	19	3	12.95	-
11	3,198,799	3,200,241	1,442	rs6578334	rs10833495	6	1	12.92	*C11orf36*
1	150,828,032	150,850,302	22,270	rs12098109	rs7526620	4	0	12.90	*LCE3C*
22	17,108,422	17,245,882	137,460	rs12484661	rs4295115	9	1	12.88	*GGT3P*
4	57,750,017	57,793,311	43,294	rs13145188	rs12500068	19	3	12.74	-
1	1,148,045	1,168,788	20,743	rs12407332	rs12093154	16	3	12.58	*SDF4, B3GALT6, FAM132A*
2	238,705,447	238,747,816	42,369	rs11695472	rs6722284	22	3	12.16	*ESPNL, KLHL30, ILKAP*
1	72,528,637	72,528,687	50	rs17526367	rs2568950	2	0	11.58	-
7	113,336	160,337	47,001	rs4096522	rs4617107	14	3	11.36	-
16	35,071,364	35,141,900	70,536	rs2219964	rs649602	8	3	11.36	-
10	41,851,973	41,918,278	66,305	rs11266039	rs12356349	9	1	11.31	-
4	946,047	1,016,866	70,819	rs6599390	rs10012074	53	3	10.86	*DGKQ, SLC26A1, IDUA, FGFRL1*

Supplementary references

1. Rutter M, Le Couteur A, Lord C (2003): *Autism Diagnostic Interview-Revised (ADI-R). Manual*. Los Angeles, CA: Western Psychological Services.
2. Lord C, Rutter M, DiLavore PC, Risi S (2001): *Autism diagnostic observation schedule. Manual*. Los Angeles, CA: Western Psychological Services.
3. Szatmari P, Paterson AD, Zwaigenbaum L, Roberts W, Brian J, Liu XQ, *et al.* (2007): Mapping autism risk loci using genetic linkage and chromosomal rearrangements. *Nat Genet* 39:319-328.
